# A nested randomised controlled trial of a leaflet, containing information on research, to increase the recruitment rate of reform (reducing falls with orthoses and a multifaceted podiatry) trial participants

**DOI:** 10.1186/1745-6215-14-S1-O109

**Published:** 2013-11-29

**Authors:** Catherine Arundel, David Torgerson, Laura Jefferson, Sarah Cockayne

**Affiliations:** 1 University of York, York, UK

## Background

Poor recruitment is a major problem in randomised trials. Although pre-notification has been demonstrated to be an effective strategy for increasing response rates to postal questionnaires (Edwards et al, 2010), the usefulness of this approach to aid trial recruitment has never been evaluated.

This study, nested within the NIHR funded REFORM Trial, aimed to identify whether increasing understanding of research in potential trial participants, prior to being approached for consent, can increase trial recruitment.

## Methods

Prospective participants for the REFORM trial were identified by local Podiatry or Falls clinics. A pilot trial showed a 3% recruitment rate. To have 80% power (2p = 0.05) and increase recruitment to 5%, we needed to randomise 3300 participants (2:1 in favour of control arm) to receive a general leaflet about research (2 weeks prior to the REFORM consent packs being sent) or to receive no prior information. Leaflets are being mailed out in April to June 2013.

## Results

Mail out for this nested sub study will be completed by 1st July 2013. The results of the study will be presented at the Clinical Trials Methodology Conference. The main outcome is the number of participants randomised between the two arms of the study.

## Discussion

The trial findings should provide useful information about recruitment aids for researchers designing future randomised controlled trials.
